# Prognostic factors in colorectal liver metastases patients with various tumor numbers treated by liver resection: a single-center, retrospective study

**DOI:** 10.1186/s12957-022-02700-4

**Published:** 2022-07-20

**Authors:** Feng-Lin Chen, Yan-Yan Wang, Wei Liu, Bao-Cai Xing

**Affiliations:** Key Laboratory of Carcinogenesis and Translational Research, Ministry of Education, Peking University School of Oncology, Hepatopancreatobiliary Surgery Department I, Beijing Cancer Hospital and Institute, Beijing, People’s Republic of China

**Keywords:** Colorectal cancer, Liver metastasis, Hepatectomy, Risk factors

## Abstract

**Background:**

Multiple liver metastases is considered a risk factor for overall survival of colorectal liver metastases patients (CRLM) after curative resection. However, whether the prognostic factors were constant in patients with various liver metastases (LM) numbers has not been adequately investigated. This retrospective study aimed to evaluate the changing of prognostic factors on overall survival (OS) in CRLM patients with various LM after curative resection.

**Methods:**

Patients who underwent liver resection for CRLM between January 2000 and November 2020 were retrospectively studied. They were divided into three subgroups according to LM numbers by X-tile analysis. Multivariable analysis identified prognostic factors in each subgroup. Nomograms were built using different prognostic factors in three subgroups, respectively. Performance of the nomograms was assessed according to the concordance index (C-index) and calibration plots. The abilities of different scoring systems predicting OS were compared by calculating the area under the time-dependent receiver operating characteristic (ROC) curve (AUC).

**Results:**

A total of 1095 patients were included. Multivariable analysis showed tumor number increasing was an independent risk factor. Patients were subsequently divided into 3 subgroups according to the number of LM by X-tile analysis, namely solitary (*n* = 375), 2–4 (*n* = 424), and ≥ 5 (*n* = 296). The 3-year and 5-year OS rates were 64.1% and 54.0% in solitary LM group, 58.1% and 41.7% in 2–4 LM group, and 50.9% and 32.0% in ≥ 5 LM group, respectively (*p* < 0.001). In multivariable analysis, *RAS* mutation was the only constant independent risk factor in all subgroups. The nomograms were built to predict survival based on independent factors in three subgroups. The C-index for OS prediction was 0.707 (95% CI 0.686–0.728) in the solitary LM group, 0.695 (95% CI 0.675–0.715) in the 2–4 LM group, and 0.687 (95% CI 0.664–0.710) in the ≥ 5 LM group. The time-dependent AUC values of nomograms developed using different risk factors after stratifying patients by tumor number were higher than the traditional scoring systems without patient stratification.

**Conclusions:**

The prognostic factors varied among CRLM patients with different LM numbers. *RAS* mutation was the only constant risk factor. Building prediction models based on different prognostic factors improve patient stratification.

**Supplementary Information:**

The online version contains supplementary material available at 10.1186/s12957-022-02700-4.

## Introduction

Colorectal cancer (CRC) is the third most common cancer and the second leading cause of cancer-related death worldwide [1]. About 50% of patients will develop colorectal liver metastases during the course of the disease [2]. Hepatic resection is still the golden treatment to achieve long-term survival, with 5-year survival in 32–41% of patients [3, 4].

Previous studies have revealed numerous prognostic factors after curative resection of colorectal liver metastases patients (CRLM), and tumor number is one of the most important factors [5–7]. Patients with several liver metastases have been studied extensively, and multiple liver metastases presented poor biological behavior. More than four liver metastases used to be a contraindication for liver resection [8]. Fortunately, in the modern era of chemotherapy regimens, it was reported that patients with 10 or more CRLM also achieved long-term survival after liver resection [9]. Notably, patients with different tumor numbers have significantly different survival outcomes [5, 6, 10], and the prognostic factors in these patients might be different. Previous retrospective studies have reported inconsistent independent risk factors, which may be caused by the heterogeneity in clinic-pathological characteristics of patients included in different studies [6, 11–13]. As more patients with a high number of liver metastases (LM) underwent hepatic resection, analyzing patients without stratification might be unreliable.

The present study investigated the impact of the most widely used clinicopathological and surgical-related risk factors in CRLM patients with different tumor numbers. An optimal cut-off of tumor number over overall survival (OS) was calculated by X-tile analysis. After stratifying patients by tumor number, the significantly changing independent risk factors were revealed. The prognostic utility of the scoring system built after stratification was compared with traditional scoring systems without patient stratification.

## Methods

### Study population

We retrospectively included patients who underwent liver resection for CRLM between January 2000 and November 2020 at the Hepatopancreatobilary Surgery Department I of Peking University Cancer Hospital. All patients included in the study signed a written consent form. The study was examined and certified by the Ethics Committee of Beijing Cancer Hospital and performed according to the Declaration of Helsinki.

The inclusion criteria were as follows: (1) pathologically confirmed CRLM; (2) considered a resectable disease by a multidisciplinary team (MDT) before surgery; (3) no evidence of extrahepatic disease other than lung metastases; (4) complete resection of the metastases and primary site. According to the criteria, this retrospective observational study included 1095 patients.

### Disease management

Abdominal and pelvic contrast-enhanced computed tomography (CT) scan, chest CT and abdominal enhanced magnetic resonance imaging (MRI) were routinely used to assess the resectability of CRLM and detect extrahepatic disease. The surgery decision was made by MDT discussion. Resections of three or more segments were considered a major hepatic resection [14]. More than 40% of the future liver remnant was preserved in patients with chemotherapy injuries [15]. Portal vein embolization and 2-stage hepatectomy were used in patients with insufficient remnant volume [16]. Patients received a complement ablation or stereotactic body radiotherapy if R2 resection. Patients routinely received perioperative chemotherapy for 6 months unless contraindicated due to poor tolerance or comorbidities. All patients were followed up every 3 months for 2 years after liver resection, then every 6 months. Follow-up included abdominal and pelvic enhanced CT, chest CT and measurement of carcinoembryonic antigen (CEA) and carbohydrate antigen 19-9 (CA19-9) levels.

### Clinicopathologic characteristics

All the preoperative prognostic factors were assessed at the time of surgery. Tumor number was obtained from preoperative imaging. OS was calculated from the date of surgery to the latest news date. All data were collected prospectively and analyzed retrospectively.

### Study design

Previous studies reported that extrahepatic disease is a strong adverse prognostic factor [6, 17, 18]. However, patients with pulmonary metastases have better outcomes [19]. To avoid masking the potential risk factors, the current study included patients with lung metastases but excluded patients with other sites of extrahepatic disease.

For the first stage of analysis, the multivariable analysis revealed that increasing tumor number was an independent risk factor. Then, the optimal cut-off of tumor numbers was established by X-tile analysis. For the second analysis stage, patients were subsequently divided into three subgroups, and multivariable analysis was done to identify independent risk factors. For the third analysis stage, the prognostic utility of scoring systems built after stratifying patients by tumor number was compared to traditional scoring systems built without stratification.

### Calculation of the risk score by Fong’s Clinical Risk Score, and scoring systems proposed by Nordlinger et al., Brudvik et al., Konopke et al., and Nagashima et al.

The score was calculated by assigning one point to each criterion in the following scoring systems. Criterions of Fong’s Clinical Risk Score: positive nodal status of primary, disease-free interval < 12 months, number of LM > 1, preoperative CEA > 200 ng/ml, and size of the largest tumor > 5 cm [6]. Criterions of Nordlinger et al.: age > 60, T4 disease of the primary tumor, disease-free interval < 24 months, > 3 liver metastases, size of the largest tumor > 5 cm, lymphatic spread of primary cancer, and positive margin [20]. Criterions of Brudvik et al.: *RAS* mutation, positive nodal status of primary, size of the largest tumor > 5 cm [11]. Criterions of Konopke et al.: synchronous disease, > 3 liver metastases, and CEA > 200 ng/ml [21]. The scores of the scoring system proposed by Nagashima et al. were calculated by the subsequent formula: *y* = 0.8057 × serosal invasion of primary, 1; (−), 0 + 0.8375 × positive nodal status of primary, 1; (−), 0 + 0.8609 × number of LM > 1, 1; solitary, 0 + 1.4532 × size of the largest tumor > 5 cm, 1; ≦ 5 cm, 0 + 1.3957 × extrahepatic metastases. (+), 1; (−), 0 [22].

### Statistical analysis

Continuous variables were transformed into categorical data by using the cut-off adapted from the previous literature [6, 11, 23]. Categorical variables were summarized as frequency and percentage and were compared using the chi-square test. The X-tile 3.6.1 software (Yale University, New Haven, CT, USA) was used to determine the cut-off value of tumor number and stratify subgroups of patients identified according to the OS. The survival probabilities were calculated according to the Kaplan-Meier method, and survival plots were compared with the log-rank test. Missing data assumed to be missing at random were imputed by multiple imputations [24]. Univariable and multivariable analyses of clinicopathological factors were performed by Cox’s proportional hazard model to identify independent prognostic factors for OS. Nomograms were built based on the results of the multivariable analysis. The predictive accuracy of the nomograms was measured by the concordance index (C-index) and assessed by calibration. The abilities of different scoring systems to predict OS were compared by calculating the area under the time-dependent receiver operating characteristic (ROC) curve (AUC). A two-tailed *p* value less than 0.05 was considered to be statistically significant. The analysis was done using SPSS 27.0 (SPSS Inc., IBM, Chicago, IL) and R, version 4.1.0 (www.r-project.org).

## Results

### Selection of optimal tumor number cut-off

**Fig. 1:**
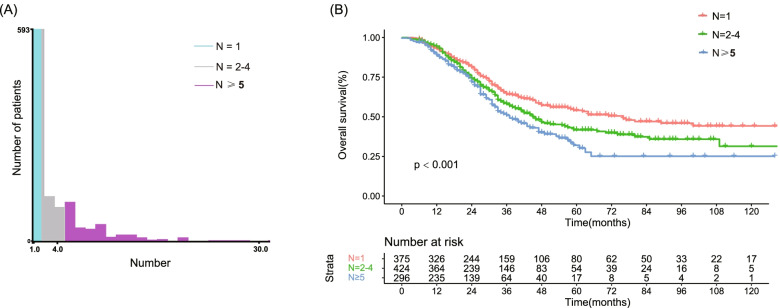
X-tile analysis was used to determine the optimal cut-off value for tumor number identified according to OS. **A** The optimal cut-off value was identified as 1 and 4. **B** The OS rate between the three groups. Abbreviations: OS: overall survival

In the present study, 1095 patients who met the inclusion criteria were included. Multivariable analysis revealed that increasing tumor number was an independent risk factor for OS (HR = 1.025, 95%CI 0.997–1.053, *p* = 0.043). The X-tile analysis determined that LM ≥ 2 and LM ≥ 5 was the optimal cut-off value to predict OS (Fig. 1A). Thus, patients were divided into three groups in the subsequent analysis: solitary LM, 2–4 LM, and ≥ 5 LM.

### Clinicopathologic characteristics

The clinicopathologic characteristics of patients in three subgroups were summarized in Table 1. *RAS* mutation was found in 155 (41.33) patients in solitary LM, 168 (39.62) patients in 2–4 LM, and 126 (42.57) patients in ≥ 5 LM. No perioperative deaths occurred within 30 days.

**Table 1:** Baseline characteristics in patients with solitary LM, 2–4 LM, ≥ 5 LM

Characteristic	*N* = 1	*N* = 2–4	*N* ≥ 5	*p* value
	(*n* = 375)	(*n* = 424)	(*n* = 296)	
Gender				
Male	243 (64.80)	264 (62.26)	202 (68.24)	0.255
Female	132 (35.20)	160 (37.74)	94 (31.76)	
Age				
< 60	195 (52.00)	218 (51.42)	193 (65.20)	< 0.001
≥ 60	180 (48.00)	206 (48.58)	103 (34.80)	
Primary site				
Left	304 (81.07)	352 (83.02)	244 (82.43)	0.766
Right	71 (18.93)	72 (16.98)	52 (17.57)	
Primary tumor T stage				
T_1−2_	39 (10.40)	34 (8.02)	20 (6.76)	0.220
T_3−4_	336 (89.60)	390 (91.98)	276 (93.24)	
Primary tumor lymph node status				
Negative	129 (34.40)	133 (31.37)	78 (26.35)	0.081
Positive	246 (65.60)	291 (68.63)	218 (73.65)	
*RAS* mutation	155 (41.33)	168 (39.62)	126 (42.57)	0.722
Preoperative chemotherapy				
No	182 (48.53)	72 (16.98)	14 (4.73)	< 0.001
Yes	193 (51.47)	352 (83.02)	282 (95.27)	
Disease-free interval				
≥ 12 m	134 (35.73)	59 (13.92)	11 (3.72)	< 0.001
< 12 m	241 (64.27)	365 (86.08)	285 (96.28)	
Bilobar distribution	27 (7.2)	260 (61.32)	269 (90.88)	< 0.001
Maximum tumor diameter ≥ 5 cm	66 (17.60)	49 (11.56)	22 (7.43)	< 0.001
Pulmonary metastases	42 (11.20)	50 (11.79)	31 (10.47)	0.859
Margin status				
R0	344 (91.73)	363 (85.61)	214 (72.30)	
R1	29 ( 7.73)	56 (13.21)	72 (24.32)	< 0.001
R2	2 ( 0.53)	5 ( 1.18)	10 ( 3.38)	
Hepatic resection				
Minor	338 (90.13)	339 (79.95)	171 (57.77)	< 0.001
Major	37 (9.87)	85 (20.05)	125 (42.23)	
Intraoperative ablation	9 (2.40)	76 (17.92)	133 (44.93)	< 0.001
Simultaneous resection				
No	305 (81.33)	321 (75.71)	229 (77.36)	0.149
Yes	70 (18.67)	103 (24.29)	67 (22.64)	
CEA level (ng/ml)				
< 200	366 (97.60)	409 (96.46)	290 (97.97)	0.419
≥ 200	9 (2.40)	15 (3.54)	6 (2.03)	
CA19-9 level (IU/ml)				
< 50	278 (74.13)	330 (77.83)	224 (75.68)	0.470
≥ 50	97 (25.87)	94 (22.17)	72 (24.32)	
Perioperative RBC transfusion	19 (5.07)	33 (7.78)	33 (11.15)	0.014

*Abbreviations*: *LM* liver metastases, *RAS* rat sarcoma viral oncogene homolog, *CEA* carcinoembryonic antigen, *CA19-9* carbohydrate antigen 19-9, *RBC* red blood cell

### Survival

The median follow-up time was 42 months. The overall survival of each group were calculated by the Kaplan-Meier method (Fig. 1B). The 1-year, 3-year, and 5-year OS rates was 93.1%, 64.1%, and 54.0% in the solitary LM group, 92.8%, 58.1%, and 41.7% in the 2–4 LM group, and 88.9%, 50.9% and 32.0% in the ≥ 5 LM group.

### Univariable and multivariable analysis of factors associated with OS

Univariable analysis of risk factors associated with OS of three groups is summarized in Table 2. Variables with *p* values < 0.15 at univariate analysis were entered into a Cox proportional hazard model for multivariable analysis. In solitary LM group, the independent prognostic factors were: right-sided primary tumor (HR = 1.724, 95%CI 1.167–2.546, *p* = 0.006), preoperative pulmonary metastasis (HR = 1.915, 95%CI 1.179–3.109, *p* = 0.009), *RAS* mutation (HR = 1.942, 95%CI 1.374–2.744, *p* = 0.000), preoperative CEA ≥ 200 ng/ml (HR = 4.444, 95%CI 2.071–9.536, *p* = 0.000), preoperative CA19-9 ≥ 50 IU/ml (HR = 2.289, 95%CI 1.593–3.289, *p* = 0.000). In patients with 2–4 LM, the independent risk factors were primary tumor stage T_3-4_ (HR = 3.851, 95%CI 1.689–8.781, *p* = 0.001), primary tumor LN positive (HR = 1.670, 95%CI 1.183–2.358, *p* = 0.004). maximum tumor diameter ≥ 5 cm (HR = 2.000, 95%CI 1.309–3.056, *p* = 0.001), preoperative pulmonary metastasis (HR = 1.711, 95%CI 1.113–2.630, *p* = 0.014), *RAS* mutation (HR = 1.456, 95%CI 1.059–2.003, *p* = 0.021), preoperative CA19-9 ≥ 50 IU/ml (HR = 1.863, 95%CI 1.313–2.642, *p* = 0.001). In patients with ≥ 5 LM, the independent preoperative risk factors were primary tumor LN positive (HR = 1.984, 95%CI 1.275–3.087, *p* = 0.002), maximum tumor diameter ≥ 5 cm (HR = 2.260, 95%CI 1.302–3.923, *p* = 0.004), and *RAS* mutation (HR = 3.150, 95%CI 2.153–4.609, *p* = 0.000). Of all the three groups, *RAS* mutation was the only constant independent risk factor, and showed the highest HR in patients with ≥ 5 CRLM. The results of multivariate analysis are summarized in Table 3.

**Table 2:** Results of univariate analysis of predictors for overall survival in three subgroups

Risk factors	*N* = 1 (*n* = 375)			*N* = 2 4 (*n* = 424)			*N* ≥ 5 (*n* = 296)		
	HR	95%CI	*p*	HR	95%CI	*p*	HR	95%CI	*p*
Age group									
< 60	Ref			Ref			Ref		
≥ 60	0.995	0.713–1.388	0.975	0.879	0.654–1.182	0.394	0.899	0.609–1.326	0.591
Gender									
Male	Ref			Ref			Ref		
Female	0.781	0.558–1.095	0.152	0.787	0.583–1.062	0.118	1.005	0.680–1.484	0.981
Primary site									
Left	Ref			Ref			Ref		
Right	1.916	1.309–2.803	0.001	1.143	0.790–1.654	0.478	1.083	0.682–1.719	0.736
Primary tumor T stage									
T_1−2_	Ref			Ref			Ref		
T_3−4_	1.396	0.754–2.584	0.289	3.239	1.433–7.323	0.005	1.622	0.713–3.693	0.249
Primary tumor lymph node status									
Negative	Ref			Ref			Ref		
Positive	1.153	0.813–1.636	0.425	1.763	1.251–2.485	0.001	1.750	1.138–2.693	0.011
Disease-free interval									
≥ 12 m	Ref			Ref			Ref		
< 12 m	1.235	0.874–1.745	0.232	1.031	0.675–1.575	0.888	0.673	0.328–1.382	0.281
Distribution of liver metastases									
Unilobar	Ref			Ref			Ref		
Bilobar	1.396	0.816–2.391	0.224	1.115	0.820–1.516	0.489	0.979	0.583–1.643	0.935
Maximum tumor diameter									
< 5 cm	Ref			Ref			Ref		
≥ 5 cm	1.343	0.901–2.004	0.148	2.189	1.491–3.213	0.000	2.564	1.486–4.426	0.001
Pulmonary metastases									
Negative	Ref			Ref			Ref		
Positive	1.839	1.142–2.962	0.012	1.759	1.150–2.690	0.009	1.702	1.003-2.888	0.049
*RAS* mutation									
Wild	Ref			Ref			Ref		
Mutation	1.973	1.412–2.757	0.000	1.602	1.188–2.162	0.002	2.913	2.011–4.219	0.000
Preoperative chemotherapy									
No	Ref			Ref			Ref		
Yes	0.916	0.656–1.279	0.607	0.893	0.618–1.290	0.547	0.948	0.462–1.947	0.885
CEA level (ng/ml)									
< 200	Ref			Ref			Ref		
≥ 200	4.179	2.041–8.557	0.000	2.162	1.140–4.102	0.018	1.081	0.341–3.427	0.895
CA19-9 level (IU/ml)									
< 50	Ref			Ref			Ref		
≥ 50	2.175	1.543–3.067	0.000	2.355	1.712–3.239	0.000	1.362	0.904–2.052	0.140
Margin status									
R0	Ref			Ref			Ref		
R1	1.201	0.664–2.171	0.544	1.237	0.820–1.864	0.310	1.070	0.720–1.590	0.737
R2	1.935	0.479–7.824	0.354	1.025	0.254–4.138	0.973	0.746	0.183–3.034	0.682
Hepatic resection									
Minor	Ref			Ref			Ref		
Major	1.324	0.797–2.199	0.279	1.298	0.908–1.856	0.152	1.190	0.826–1.714	0.351
Perioperative RBC transfusion									
No	Ref			Ref			Ref		
Yes	1.490	0.757–2.933	0.248	1.327	0.833–2.116	0.234	1.252	0.780–2.010	0.352
Intraoperative ablation									
No	Ref			Ref			Ref		
Yes	0.293	0.041–2.094	0.221	0.765	0.474–1.235	0.273	0.766	0.506–1.161	0.209
Simultaneous resection									
No	Ref			Ref			Ref		
Yes	1.385	0.914–2.097	0.125	0.871	0.615–1.232	0.434	0.843	0.549–1.294	0.434

*Abbreviations*: *LM* liver metastases, *RAS* rat sarcoma viral oncogene homolog, CEA carcinoembryonic antigen, *CA19-9* carbohydrate antigen 19-9, *RBC* red blood cell

**Table 3:** Results of multivariable analysis of predictors for overall survival in three subgroups

	Risk factors	Preoperative factors		
		HR	95%CI	*p*
*N* = 1	Primary site			
(*n* = 375)	Left	Ref		
	Right	1.724	1.167–2.546	0.006
	Pulmonary metastases			
	Negative	Ref		
	Positive	1.915	1.179–3.109	0.009
	*RAS* mutation			
	Wild	Ref		
	Mutation	1.942	1.374–2.744	0.000
	CEA level (ng/ml)			
	< 200	Ref		
	≥ 200	4.444	2.071–9.536	0.000
	CA19–9 level (IU/ml)			
	< 50	Ref		
	≥ 50	2.289	1.593–3.289	0.000
*N* = 2–4	Primary tumor T stage			
(*n* = 424)	T_1−2_	Ref		
	T_3−4_	3.851	1.689–8.781	0.001
	Primary tumor lymph node status			
	Negative	Ref		
	Positive	1.670	1.183–2.358	0.004
	Maximum tumor diameter			
	< 5 cm	Ref		
	≥ 5 cm	2.000	1.309–3.056	0.001
	Pulmonary metastases			
	Negative	Ref		
	Positive	1.711	1.113–2.630	0.014
	*RAS* mutation			
	Wild	Ref		
	Mutation	1.456	1.059–2.003	0.021
	CA19-9 level (IU/ml)			
	< 50	Ref		
	≥ 50	1.863	1.313–2.642	0.001
*N* ≥ 5	Primary tumor lymph node status			
(*n* = 296)	Negative	Ref		
	Positive	1.984	1.275–3.087	0.002
	Maximum tumor diameter			
	< 5 cm	Ref		
	≥ 5 cm	2.260	1.302–3.923	0.004
	*RAS* mutation			
	Wild	Ref		
	Mutation	3.150	2.153–4.609	0.000

*Abbreviations*: *LM* liver metastases, *RAS* rat sarcoma viral oncogene homolog, *CEA* carcinoembryonic antigen, *CA19-9* carbohydrate antigen 19-9

### Construction of prognostic nomograms in each subgroup

**Fig. 2:**
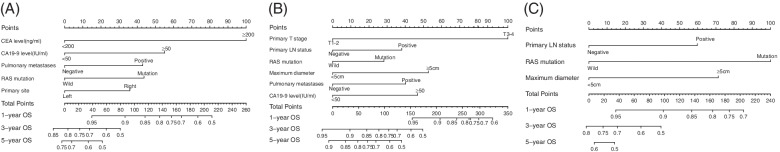
Nomograms developed from independent risk factors to predict OS in three subgroups. **A** solitary LM, **B** 2–4 LM, and **C** ≥ 5 LM. Abbreviations: OS: overall survival; LM: liver metastases

Nomograms with point scales were built according to independent prognostic factors identified in each subgroup. These independent were assigned specific scales. In patients with solitary LM, the point scales were: preoperative CEA ≥ 200 ng/ml, 100’; preoperative CA19-9 ≥ 50 IU/ml, 55’; preoperative pulmonary metastasis, 43’; *RAS* mutation, 44’; right-sided primary tumor, 36’; In patients with 2–4 LM, the point scales were: primary tumor stage T_3-4_, 100’; primary tumor LN positive, 38’; maximum tumor diameter ≥ 5 cm, 53’; preoperative pulmonary metastasis, 40’; *RAS* mutation, 28’; preoperative CA19-9 ≥ 50 IU/ml, 47’; In patients with ≥ 5 LM, the point scales were: primary tumor LN positive, 60’; maximum tumor diameter ≥ 5 cm, 71’; *RAS* mutation, 100’; The results were summarized in Supplementary Table 1. The sum of the scores of each variable was plotted on the total point axis. The estimated probabilities of OS after liver resection at 1, 3, and 5 years were obtained by drawing a horizontal line from the plotted total points axis straight to the survival axis. The results are summarized in Fig. 2. The C-index for OS prediction was 0.707 (95%CI 0.686–0.728) in the solitary LM group, 0.695 (95%CI 0.675–0.715) in the 2–4LM group, and 0.687 (95%CI 0.664–0.710) in ≥ 5 LM group. A calibration plot for survival probability at 1, 3, and 5 years demonstrated good calibration in each subgroup between the prediction by the nomogram and the actual observation (Supplementary Figure 1, Supplementary Figure 2, Supplementary Figure 3).

### Comparison of scoring systems built after stratification and other scoring systems

**Fig. 3:**
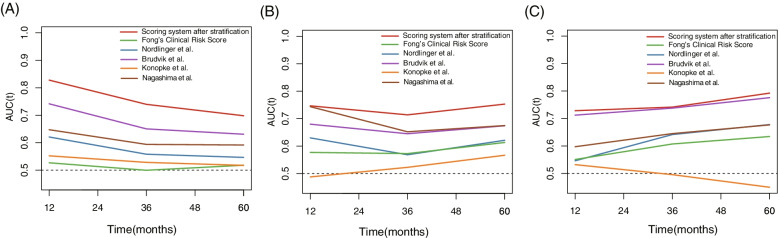
Performance of the scoring system after stratifying patients by tumor numbers. Time-dependent AUC values showed the performance of the new scoring system and other models in predicting OS in **A** solitary LM, **B** 2–4 LM, and **C** ≥ 5 LM. Abbreviations: AUC: area under the curve; OS: overall survival; LM: liver metastases

The nomogram-predicted scores were calculated in each subgroup. The AUCs of the nomogram score in predicting OS at 1-year, 3-year, and 5-year were as follows: 0.828, 0.740, and 0.700 in the solitary LM group, 0.747, 0.714, and 0.753 in the 2–4 LM group, and 0.728, 0.741, and 0.792 in the ≥ 5 LM group. The AUCs of the scoring system in predicting OS after stratifying patients into three subgroups was higher than the Fong’s Clinical Risk Score and scoring systems proposed by Nordlinger et al., Brudvik et al., Konopke et al., and Nagashima et al. The results are summarized in Fig. 3.

## Discussion

The present study revealed the change of the most widely used clinicopathological and genetic risk factors in patients with different tumor numbers. *RAS* mutation was the only constant independent risk factor among all patient groups. The scoring system developed using different risk factors in patients with different tumor numbers outperformed the commonly used scoring systems, including Fong’s Clinical Risk Score and scoring systems proposed by Nordlinger et al., Brudvik et al., Konopke et al., and Nagashima et al. Our study shed light on further risk stratification of surgically treated CRLM patients.

Liver resection has always been the golden treatment of CRLM. However, 60 to 80% of patients experience recurrence after resection, and the majority of such recurrences occur within 2 years [25, 26]. As surgeons become more proficient in the technical aspects of resection, patient selection criteria based on biologic determinants of outcome are increasingly important. Criteria are needed to ensure that patients selected for surgery benefit from such invasive procedures. Therefore, many clinical scoring systems have been developed to predict tumor recurrence and survival outcome in attempts to assist in clinical decision-making. It is necessary to maximize the benefit of individualized treatment in patients with different risk levels.

Multiple factors have been reported to be associated with survival outcomes, with tumor number being one of these [20, 27–32]. Large tumor numbers indicated poor tumor behavior. Although patient outcome changes significantly with tumor numbers, previous studies identify independent risk factors from all patients without stratification, and the results differ from studies [6, 11, 20, 27]. The difference in baseline characteristics may result in such discrepancy. Risk factors observed in one patient group may not be present in another. Also, traditional scoring systems were based on all patients [6, 11, 33]. Previous studies have questioned the prognostic utility of these scoring systems [21, 34]. The significantly changing risk factors among patients with different tumor numbers may explain their unsatisfying prognostic utility. Surgery indications expanded with the development of surgical techniques and systemic therapy. Currently, there is no numerical limit to resectability [35–37]. As more patients with high tumor numbers receive surgical treatment, applying the same scoring system in patients with various tumor numbers may reduce the predictive ability. More accurate patient stratification is required as more patients with high tumor number was treated surgically.

The present study was the first to describe the changing prognostic factors in patients with different tumor numbers. Multivariable analysis showed that tumor number was an independent risk factor for prognosis. Furthermore, X-tile analysis identified ≥ 2 and ≥ 5 as the optimal cut-off of the tumor number. After dividing patients into subgroups by the proposed cut-offs, we described the changing prognostic factors in patients with different tumor numbers. The scoring system constructed after stratifying patients outperformed the commonly used scoring systems developed without stratification. We suggested a better way to develop scoring systems with a more accurate patient stratification. Our study may light on the future improvement of risk stratification. Therefore, a more individualized treatment could be offered to CRLM patients. For example, traditionally, maximum tumor diameter ≥ 5 cm was considered a risk factor. However, it is not an independent risk factor in patients with a single LM. For this patient subgroup, maximum tumor diameter ≥ 5 cm should not affect treatment decisions.

*RAS* mutation has been identified as an adverse factor for overall survival in the previous literature [38]. In our study, *RAS* mutation is the only independent risk factor that constantly exists. Previous studies revealed that *RAS* mutation confers a constant, moderate risk of decreased survival [39]. These results suggested molecular profiling might be a steadier prognostic factor than clinicopathologic factors. A recent study reported that extended molecular profiling could further improve patient stratification and provide a highly prognostic scoring system [40]. As more cancer-related gene mutations were identified, molecular profiling might play a more important role in further improving scoring systems. Our results also showed that *RAS* mutation had the highest hazard ratio in patients with ≥ 5 liver metastases, implying that *RAS* mutation may confer a higher risk in patients with a higher disease burden. Careful monitoring and follow-up should be taken if *RAS* mutation was detected in these patients.

Several limitations of this study should be considered. While we included a large cohort of patients, the median follow-up time was relatively short. All patients included in this study were from a single-center, which may induce selection bias. Also, the utility of building nomograms after stratification needs to be further validated by external validation of data from other centers. *BRAF* mutation and microsatellite instability were not included in this study due to their relatively low prevalence in CRLM patients treated by liver resection [41–43]. Parenchymal R1 margin was reported to be an independent risk factor for OS, while the vascular R1 margin was not [44]. Since our study ranged from 2000 to 2020, and this problem has only been fully recognized in recent years, we did not distinguish them in survival analysis due to difficulty in obtaining reliable data. Also, systematic treatment has profoundly improved in this period. Therefore, the chemotherapy details and chemotherapy response were not included in the analysis. A further prospective study on the indication and regimen of perioperative chemotherapy and the proper timing of surgery is needed.

Although prognostic factors of CRLM patients who underwent liver resection have been widely studied, the change of prognostic factors with tumor number has not been explored before. Our work may help shed light on better risk stratification of surgically treated CRLM patients and allow a stratified follow-up strategy.

## Conclusions

The impact of genetic and clinicopathological factors on OS changed over tumor number. *RAS* mutation was the only constant risk factor. After stratifying patients by tumor number, the prediction models based on different prognostic factors outperformed the commonly used scoring systems without patient stratification. An individualized risk assessment should be applied to CRLM patients with different LM numbers.

## Supplementary Information

**Additional file 1: Supplementary Figure 1.** Calibration curve for predicting OS at 1-year (A), 3-year (B), and 5-year (C) in patients with solitary LM. Abbreviations: OS: overall survival; LM: liver metastases.

**Additional file 2: Supplementary Figure 2.** Calibration curve for predicting OS at 1-year (A), 3-year (B), and 5-year (C) in patients with 2-4 LM. Abbreviations: OS: overall survival; LM: liver metastases.

**Additional file 3: Supplementary Figure 3.** Calibration curve for predicting OS at 1-year (A), 3-year (B), and 5-year (C) in patients with ≥ 5 LM. Abbreviations: OS: overall survival; LM: liver metastases.

**Additional file 4: Supplementary Table 1.** The point scales of nomograms in patients with solitary LM, 2-4 LM, ≥ 5 LM.

## Data Availability

Data are available from the corresponding author upon reasonable request.

## Abbreviations

AUC: Area under the curve
CA19-9: Carbohydrate antigen 19-9
CEA: Carcinoembryonic antigen
CEUS: Contrast-enhanced ultrasound
CI: Confidence interval
C-index: Concordance index
CRC: Colorectal cancer
CRLM: Colorectal liver metastases
CRS: Clinical risk score
CT: Computed tomography
HR: Hazard ratio
LM: Liver metastases
LN: Lymph node
MDT: Multidisciplinary team
MRI: Magnetic resonance imaging
NED: No evidence of disease
OS: Overall survival
*RAS*: Rat sarcoma viral oncogene homolog
RBC: Red blood cell
ROC: Receiver operating characteristics
