# Supplementary material for: Prognostic factors in colorectal liver metastases patients with various tumor numbers treated by liver resection: a single-center, retrospective study
Source: World J Surg Oncol. 2022 Jul 20;20:237. doi: 10.1186/s12957-022-02700-4 (PMC9297581; doi:10.1186/s12957-022-02700-4)
Supplement: Supplementary file 4 — Additional file 4: Supplementary Table 1. The point scales of nomograms in patients with solitary LM, 2-4 LM, ≥ 5 LM. [file 12957_2022_2700_MOESM4_ESM.docx]

**Supplementary Table 1**. The point scales of nomograms in patients with solitary LM, 2-4 LM, ≥ 5 LM.

| Risk factors |  | Point scales |  |
| --- | --- | --- | --- |
|  | N = 1 | N = 2-4 | N ≥ 5 |
| CEA ≥200 ng/ml | 100 | NA | NA |
| CA19-9 ≥50 IU/ml | 55 | 47 | NA |
| pulmonary metastasis | 43 | 40 | NA |
| *RAS* mutation | 44 | 28 | 100 |
| right-sided primary tumor | 36 | NA | NA |
| primary tumor stage T_3-4_ | NA | 100 | NA |
| primary tumor LN positive | NA | 38 | 60 |
| maximum tumor diameter ≥5cm | NA | 53 | 71 |

Abbreviations: LM, liver metastases; *RAS*, rat sarcoma viral oncogene homolog; CEA, carcinoembryonic antigen; CA19-9, carbohydrate antigen 19-9; LN, lymph node.
